# Supplementary material for: Identification of photoperiod-induced specific miRNAs in the adrenal glands of Sunite sheep (Ovis aries)
Source: Front Vet Sci. 2022 Jul 22;9:888207. doi: 10.3389/fvets.2022.888207 (PMC9354845; doi:10.3389/fvets.2022.888207)
Supplement: Supplementary file 1 [file Data_Sheet_1.ZIP › Supplementary file/miRDeep2 file.zip!/miRDeep2 file/Know_Structure/LP42A3/oar-mir-19b.pdf]

miRBase precursor : oar-mir-19b
Total read count : 2912
oar-miR-19b read count : 2901
remaining reads : 11

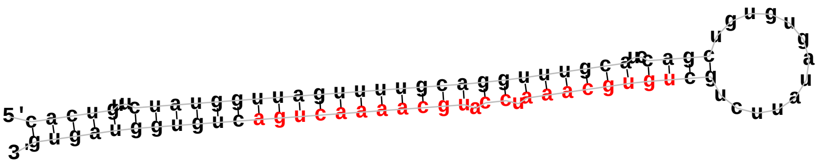

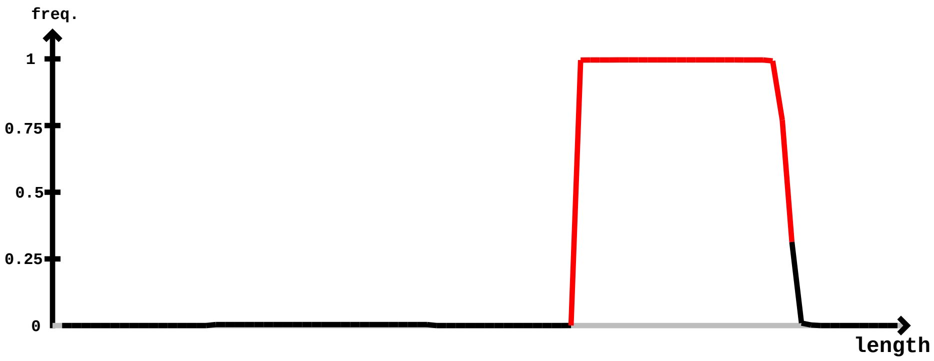

oar-miR-19b

```
5'- cacuguucuaugguuaguuuugcagguuugcauccagcugugugauauucugcugugcaaauccaugcaaaacugacugugguagug   -3'    exp
    (((((..(((((((((((((((((((((((((..((((.............)))))))))).)).))))))))))))))))))))))    reads      mm        sample
    ...............aguuuugcagguuugcauccagc.................................................    11         0         seq
    ....................................................cugugcaaauccaugcaaaacuga...........    1          0         seq
    .....................................................ugugcaaauccaugcaa.................    1          0         seq
    .....................................................ugugcaaaucUaugcaaaac..............    1          1         seq
    .....................................................ugugcaaauccaugcaaaac..............    9          0         seq
    .....................................................ugugcaaauccaugcaaaacu.............    583        0         seq
    .....................................................ugugcaaaucUaugcaaaacu.............    63         1         seq
    .....................................................ugugcaaaucUaugcaaaacug............    98         1         seq
    .....................................................ugugcaaauccaugcaaaacug............    1232       0         seq
    .....................................................ugugcaaauccaugcaaaacuga...........    775        0         seq
    .....................................................ugugcaaaucUaugcaaaacuga...........    109        1         seq
    .....................................................ugugcaaauccaugcaaaacugaU..........    20         1         seq
    .....................................................ugugcaaauccaugcaaaacugac..........    1          0         seq
    .....................................................ugugcaaauccaugcaaaacugacu.........    1          0         seq
    .....................................................ugugcaaauccaugcaaaacugaUu.........    5          1         seq
    ......................................................gugcaaauccaugcaaaacuga...........    1          0         seq
    .........................................................caaaucUaugcaaaacu.............    1          1         seq
```
